# Percutaneous pharmacomechanical thrombectomy using AngioJet® for acute limb ischaemia due to infective endocarditis: a case report

**DOI:** 10.1093/ehjcr/ytag275

**Published:** 2026-05-11

**Authors:** Muhamad Taufik Ismail, Helvina Vika Etami, Balqis Khoirunnisa, Caroline Shania Santoso, Dyah Wulan Anggrahini

**Affiliations:** Department of Cardiology and Vascular Medicine, Faculty of Medicine, Public Health, and Nursing, Gadjah Mada University, Radioputero Building 2nd Floor, Kesehatan Street No.1, Mlati, Sleman, Special Region of Yogyakarta 55281, Indonesia; Department of Cardiology and Vascular Medicine, Faculty of Medicine, Public Health, and Nursing, Gadjah Mada University, Radioputero Building 2nd Floor, Kesehatan Street No.1, Mlati, Sleman, Special Region of Yogyakarta 55281, Indonesia; Department of Cardiology and Vascular Medicine, Faculty of Medicine, Public Health, and Nursing, Gadjah Mada University, Radioputero Building 2nd Floor, Kesehatan Street No.1, Mlati, Sleman, Special Region of Yogyakarta 55281, Indonesia; Department of Cardiology and Vascular Medicine, Faculty of Medicine, Public Health, and Nursing, Gadjah Mada University, Radioputero Building 2nd Floor, Kesehatan Street No.1, Mlati, Sleman, Special Region of Yogyakarta 55281, Indonesia; Department of Cardiology and Vascular Medicine, Faculty of Medicine, Public Health, and Nursing, Gadjah Mada University, Radioputero Building 2nd Floor, Kesehatan Street No.1, Mlati, Sleman, Special Region of Yogyakarta 55281, Indonesia

**Keywords:** Acute limb ischaemia, Septic emboli, Infective endocarditis, Pharmacomechanical thrombectomy, AngioJet, Case report

## Abstract

**Background:**

Acute limb ischaemia (ALI) is a potential thromboembolic complication of infective endocarditis (IE), which requires immediate attention to prevent permanent ischaemic injury and loss of limb. Given the complex haemostatic dysregulation inherent to IE, balancing the thrombotic and haemorrhagic risk is critical in its management.

**Case summary:**

This study follows a patient diagnosed with ALI Rutherford IIa due to infective endocarditis from *Streptococcus sanguinis* infection. The patient was successfully treated with percutaneous mechanical thrombectomy using AngioJet®. Angio graphic evaluation showed restored distal arterial flow with no residual thrombus. At follow-up, the patient had returned to their daily activities without complaints.

**Discussion:**

ALI secondary to IE is traditionally treated with surgical embolectomy. However, endovascular techniques have emerged as an increasingly preferred first-line therapy, offering comparable clinical efficacy to open surgery alongside enhanced postoperative recovery. To our knowledge, this case represents the first reported use of AngioJet® percutaneous pharmacomechanical thrombectomy for ALI caused by infective endocarditis. Despite the success observed in this particular patient, this approach should remain limited to carefully selected cases until further evidence on its safety and efficacy is available.

Learning points

- Pharmacomechanical thrombectomy may be considered as an alternative to open embolectomy for IE-related ALI patients with a viable to marginally threatened limb.
- Patient safety must be prioritized when deciding to use adjunctive thrombolytics in IE-related ALI, requiring strict bleeding risk assessment and tailored dosage adjustments.
- Optimal patient outcomes are contingent upon careful patient selection and vigilant monitoring for device-related complications, such as distal embolization and haemolysis.

## Introduction

Infective endocarditis (IE) is a condition of dysregulated immunothrombosis, presenting with competing thrombotic and bleeding risk.^1^ Balancing these risks is pivotal in improving patient outcomes. Timely recognition and intervention for thromboembolic complications in IE is critical in preventing irreversible tissue necrosis and limb loss. Acute limb ischaemia (ALI) secondary to IE is traditionally treated with surgical embolectomy. However, recent studies have found that endovascular interventions achieve comparable limb salvage while reducing length of stay and improving postoperative quality of life.^2,3^

Rheolytic aspiration thrombectomy (AngioJet®) is a minimally invasive thrombus removal technique that uses a high-pressure saline jet to fragment and aspirate thrombus by the Bernoulli effect. Coupling this mechanical action with a low-dose thrombolytic bolus constitutes pharmacomechanical thrombectomy (PMT).^4,5^ As vegetations in IE are composed not only of fibrin but also bacteria, inflammatory cells, and platelets, this mechanical fragmentation and aspiration provides more effective source control than pharmacologic lysis alone.^6,7^ To date, two cases have reported the successful use of AngioJet® PMT in IE for debulking vegetations on the tricuspid valve and septic thrombophlebitis^6,8^; however, its use in IE-related ALI has not been previously published.

## Summary figure

**Figure:**
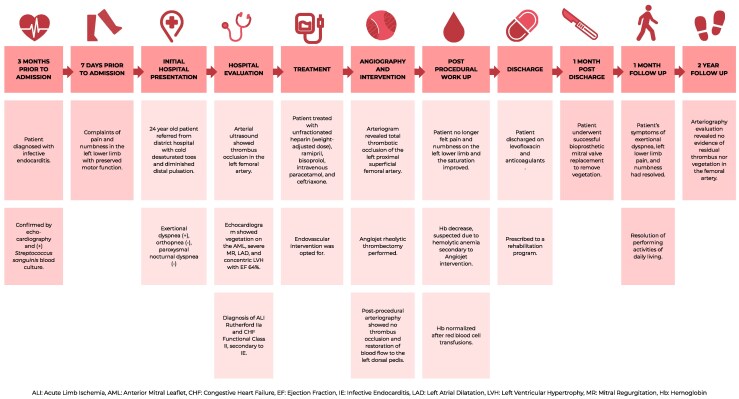


## Case presentation

A 24-year-old female was referred with a 7-day history of pain and numbness in the left lower limb with preserved motor function. Additionally, she reported exertional dyspnoea during heavy activities. She denied anginal symptoms, trauma, or environmental exposures. Medical history was negative for cardiovascular risk factors including hypertension, diabetes, and dyslipidaemia. Social history excluded tobacco and intravenous drug abuse. Family history was otherwise unremarkable. Three months prior to admission, the patient was diagnosed with definite infective endocarditis (IE), confirmed by echocardiography and positive blood cultures for *Streptococcus sanguinis*. Previous treatment details were unavailable.

Physical examination of the left lower limb revealed cold and desaturated toes: Digits I (80%), II (90%), III (60%), IV (68%), and V (88%). Pulsations were diminished from the left popliteal to dorsal pedis artery with prolonged capillary refill time and intact motor function. A pan-systolic murmur (grade 3/6) was heard at the apex. No signs of congestion, inguinal lymphadenopathy, or cutaneous manifestations of IE (e.g. Janeway lesions or Osler nodes) were present.

**Figure 1:**
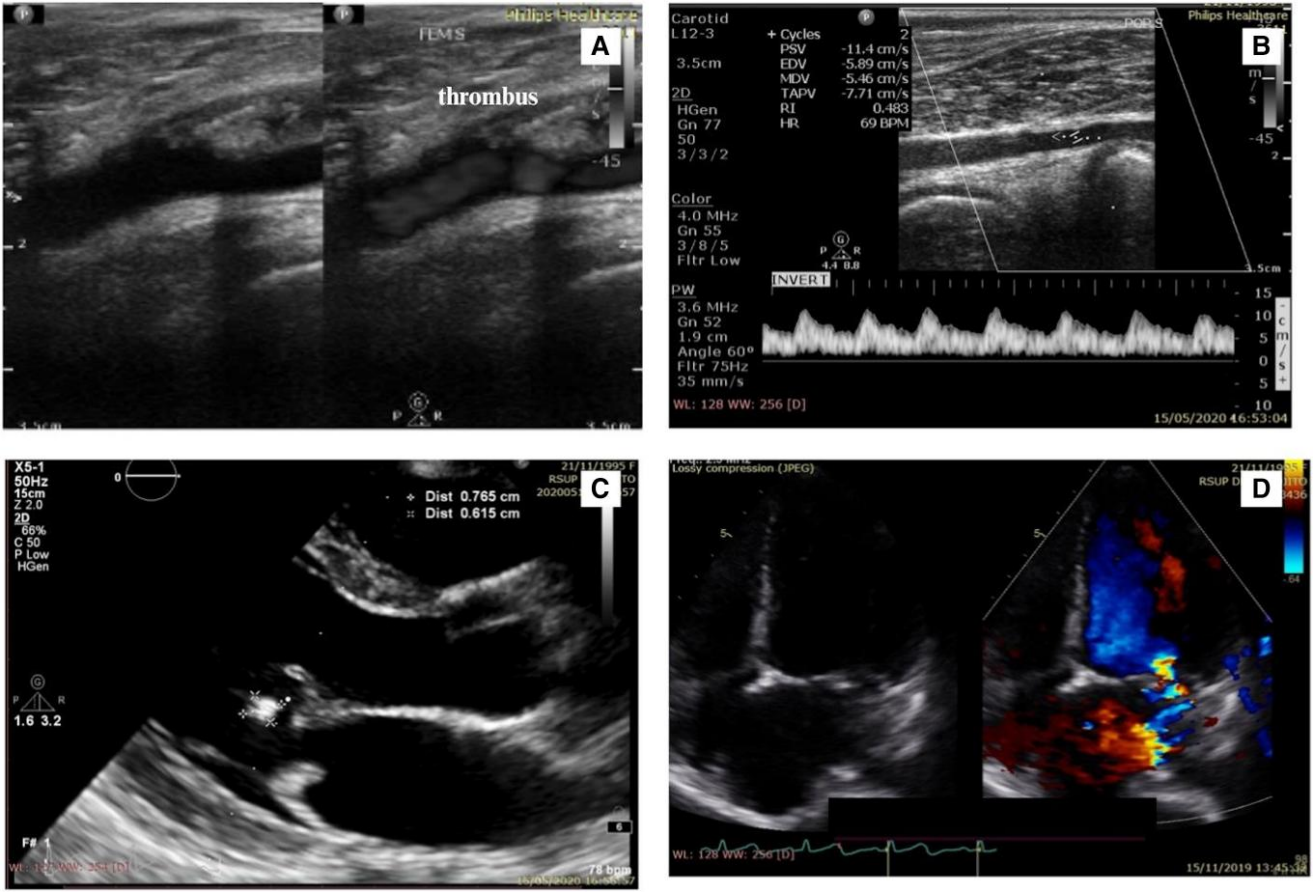
*(A)* Arterial ultrasound showed thrombus at left femoral artery. *(B)* Monophasic Doppler wave at left popliteal artery. *(C)* Echocardiography showed vegetation on anterior mitral leaflet. *(D)* Mitral regurgitation with left atrium dilatation and concentric left ventricle hypertrophy.

Chest X-ray and electrocardiogram were normal. Laboratory tests revealed mild anaemia (Hb 10.6 g/dl), leukocytosis (15.64 × 10^3^/μl), and thrombocytosis (516 × 10^3^/μl). Arterial ultrasound showed thrombus occlusion in the left femoral artery (*Figure 1A*) causing monophasic Doppler waveform in the distal popliteal artery (*Figure 1B*). Echocardiogram showed a 7 × 6 mm vegetation on anterior mitral leaflet (*Figure 1C*), severe mitral valve regurgitation, left atrium dilatation (*Figure 1D*), and concentric left ventricular hypertrophy with an ejection fraction of 64%. The vegetation size remained relatively stable compared to the initial imaging done 6 months prior (9 × 4.7 mm).

**Figure 2:**
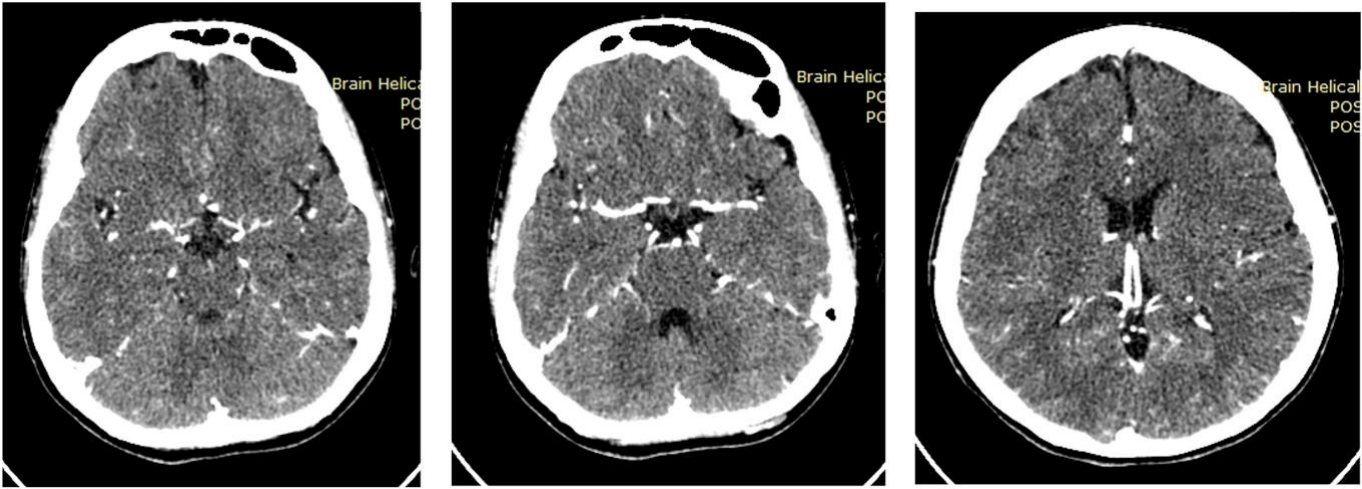
Cerebral CT angiography showed neither abnormalities nor cerebral aneurysms.

**Figure 3:**
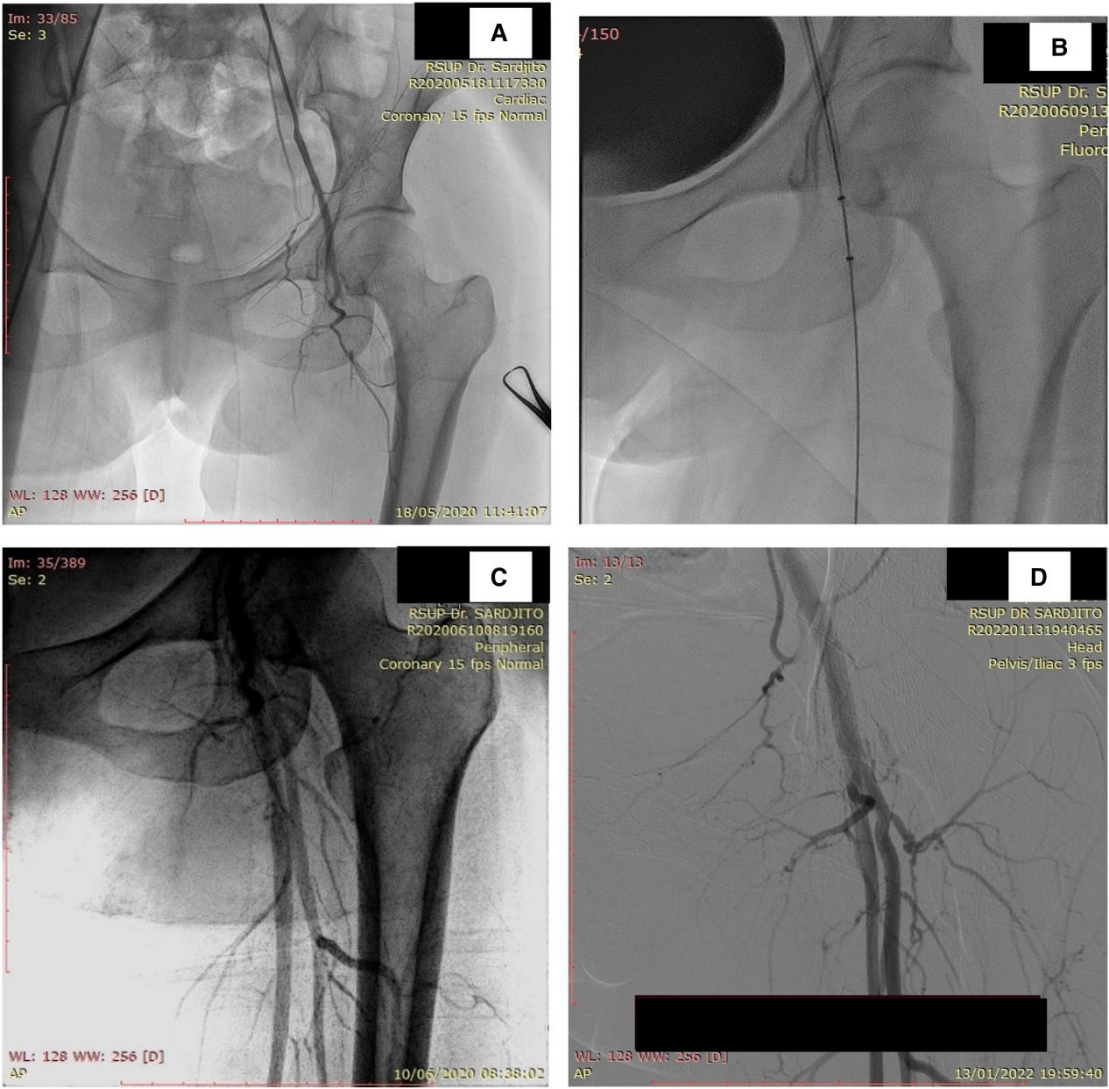
*(A)* Arteriography showed total occlusion at the left superficial femoral artery. *(B)* Pharmacomechanical thrombectomy was done at the occluded arteries. *(C)* Arteriography evaluation directly after the procedure. *(D)* Arteriography evaluation 2 years after the procedure by digital subtraction angiography.

The patient was diagnosed with ALI Rutherford IIa and congestive heart failure functional class II, both suspected secondary to IE. Weight-adjusted unfractionated heparin, ramipril, bisoprolol, paracetamol, and culture-directed ceftriaxone were administered. Cerebral computed tomography angiography (CTA) prior to percutaneous intervention was conducted to exclude IE-related cerebral aneurysms (*Figure 2*). Arteriogram revealed total thrombotic occlusion of the left proximal superficial femoral artery (*Figure 3A*), left popliteal artery vascularization was supplied by collateral artery from the profunda, and absent flow in the left anterior and posterior tibial and dorsalis pedis arteries.

Pre-dilation with 3.5 × 20 mm balloon angioplasty was performed to macerate the thrombus, followed by pulse-spray 20 mg alteplase bolus using the AngioJet® Solent Omni 6 F catheter. After 20 min, aspiration thrombectomy was performed with the total run time of 320 s (*Figure 3B*). Post-procedural arteriography showed complete thrombus removal and restored perfusion to the left dorsal pedis artery (*Figure 3C*). Continuous heparinization was maintained, and the patient was monitored in the cardiovascular care unit. Clinically, limb pain and numbness had resolved, and distal oxygen saturation improved to 95–100%. However, the patient’s post-procedural tests revealed decreased haemoglobin (7.1 g/dL), red blood cell (3.99 × 10^6^/μl from 5.89 × 10^6^/μl), and haematocrit (24.3% from 36.4%). No bleeding or haematuria was noted, and renal function remained stable, suggesting haemolytic anaemia secondary to AngioJet® intervention. Haemoglobin level normalized after red blood cell transfusion.

The patient was discharged on levofloxacin and anticoagulants, and began rehabilitation. One month later, she underwent successful bioprosthetic mitral valve replacement to remove the vegetation. Antibiotics were discontinued after surgery. At the 4-week follow-up, exertional dyspnoea, left lower limb pain, and numbness had resolved. Arteriography was performed 2 years later to evaluate residual thrombus and potential aneurysms. No evidence of thrombus or vegetation was found in the femoral artery (*Figure 3D*).

## Discussion

This case presents the successful treatment of IE-related ALI using percutaneous pharmacomechanical thrombectomy (PMT) with the AngioJet® device. The procedure restored distal arterial flow and resulted in full functional recovery for this patient. Device selection was driven by the need for rapid, safe, and efficacious intervention, tailored to the available resources at our centre. The patient presented with Rutherford IIa acute limb ischaemia without motor deficit, allowing consideration of endovascular therapy.^9^ Endovascular revascularization has emerged as an increasingly preferred first-line therapy for ALI patients, offering comparable clinical efficacy to open surgery while significantly reducing in-hospital mortality, long-term mortality, and hospital length of stay.^2,3^ Moreover, percutaneous approaches circumvent the consultative delays frequently associated with open embolectomy, facilitating the immediate revascularization required to preserve limb viability. Crucially, angiography revealed a large embolic burden occluding the proximal superficial femoral artery with collateral-dependent popliteal perfusion. Recognizing that aspiration-only thrombectomy is less likely to achieve complete and rapid revascularization, PMT using AngioJet® was therefore selected to enable efficient thrombus debulking while minimizing systemic thrombolytic exposure.^2,10^

Current ESC guidelines explicitly recommend against systemic thrombolytic therapy for IE-related stroke due to the catastrophic risk of haemorrhagic transformation. However, specific protocols for managing ALI with locally administered thrombolytics in this context remain undefined. As broader ESC recommendations caution against thrombolytic therapies in IE without compelling secondary indications, our use of AngioJet® represents a cautious deviation from these general principles, safeguarded by comprehensive risk assessment and mitigation. Prior reports have shown that the AngioJet® can be performed safely in ALI patients, making it a viable alternative to surgery in selected cases.^11^

The approach to safety for applying adjunctive thrombolytics after mechanical debulking was two-fold. First, bleeding risk was assessed by pre-procedural cerebral CTA to exclude intracranial vascular abnormalities such as mycotic aneurysms. Stable haemodynamics and lack of recent neurological symptoms further supported its safety.^4,10^ Second, alteplase was administered as a highly targeted, low-dose intrathrombus bolus rather than prolonged infusion. This approach was guided by established PMT protocols, which have demonstrated higher success rates with lower incidences of major bleeding when compared to the traditional catheter-directed thrombolysis (CDT).^11,12^ While previous studies of PMT in ALI report utilizing alteplase doses ranging from 10–25 mg,^11,12^ the 20 mg dose selection for this patient was deliberated based on the patient’s young age and relatively robust baseline health. However, future studies exploring the dose-dependent safety and efficacy profiles for PMT are warranted to establish standardized protocols.

An unexpected yet recognized complication of AngioJet® is haemolytic anaemia, caused by erythrocyte destruction during prolonged device run time.^13^ In this case, the 320-s device run time likely contributed to haemolysis, marked by post-procedural drop in haemoglobin, even though this time frame was still within the manufacturer’s recommendations of 480 s.^14^ While the high-pressure jet spray facilitates efficient thrombus fragmentation, it can also damage normal erythrocytes, leading to intravascular haemolysis.^8^ This complication was successfully managed conservatively with red blood cell transfusion.

Moreover, mechanical thrombectomy may cause distal embolization of infected material, potentially leading to microvascular occlusion or recurrent ischaemia. In such events, complementary endovascular aspiration thromboembolectomy can be performed as a salvage technique. An array of distal embolic protection devices (filters) has been developed; however, their use is not yet established as a standard of care.^9^ Beyond procedural complications, we must acknowledge that endovascular treatment does not provide definitive source control, and recurrent embolization remains possible until surgical valve intervention is performed. These risks underscore the importance of close surveillance and timely definitive valve surgery following limb revascularization.^7,10^

A notable limitation of this case is the lack of tissue confirmation, which restricts the ability to establish definitive causal inference. While IE was the presumed embolic source based on the clinical context and echocardiographic evidence, microbiological and histopathological confirmation from the femoral artery were not obtained.^7^ Consequently, a non-septic thromboembolic source cannot be entirely excluded, and the aetiology of occlusion remains presumed rather than proven. Nevertheless, this clinical presumption remains strongly supported by the complete absence of alternative embolic sources, such as atrial fibrillation, aortic aneurysms, or significant atherosclerotic disease.

## Conclusion

To our knowledge, this case represents the first reported use of AngioJet® percutaneous pharmacomechanical thrombectomy for ALI caused by infective endocarditis. Despite the success observed in this particular patient, this approach should remain limited to carefully selected cases until further evidence on its safety and efficacy are available.

## Lead author biography


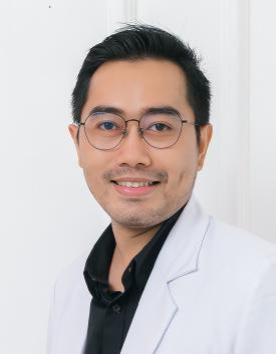
Dr. Muhamad Taufik Ismail is a cardiologist and interventionalist with a subspecialty focus in vascular medicine at Dr. Sardjito General Hospital and the Department of Cardiology and Vascular Medicine, Faculty of Medicine, Public Health, and Nursing, Universitas Gadjah Mada, Indonesia. Since 2016, he has been actively involved in comprehensive cardiovascular and endovascular care, performing procedures including PTA, vascular stenting, CDT, EVLA, TEVAR, EVAR, carotid and renal interventions, as well as coiling and embolization. He holds a doctoral degree and has published in reputable peer-reviewed journals, with research interests in vascular disease and innovative therapeutic approaches.

## Data Availability

The data underlying this article will be shared on reasonable request to the corresponding author.
